# Immune mechanisms underlying endothelial and placental dysfunction in leptospirosis during pregnancy

**DOI:** 10.3389/fcimb.2026.1952734

**Published:** 2026-10-02

**Authors:** Cecilia Nieves, Elsa-Patricia Rondon, Aindrila Kazi, Mariane Bertagnolli

**Affiliations:** 1 Cardiovascular Health Across the Lifespan Program, Research Institute of the McGill University Health Centre, Montreal, QC, Canada; 2 School of Physical and Occupational Therapy, Faculty of Medicine and Health Sciences, McGill University, Montreal, QC, Canada

**Keywords:** endothelial injury, leptospirosis, one health, placental dysfunction, preeclampsia-like syndrome, pregnancy, reproductive outcomes, zoonotic disease

## Abstract

Leptospirosis is a zoonotic disease caused by pathogenic bacteria of the genus *Leptospira* and represents a major public health concern worldwide. Although its systemic manifestations are well documented, the consequences of leptospirosis for reproduction and pregnancy, particularly in humans, remain poorly understood. This narrative review summarizes the principal reproductive outcomes associated with leptospirosis and examines the mechanisms that may contribute to pregnancy complications. In livestock, leptospirosis is a well-established cause of reproductive failure, including abortion and infertility, and is frequently associated with persistent infection and colonization of the genital tract. In humans, where available evidence remains limited, adverse pregnancy outcomes may be mediated predominantly by systemic endothelial injury, inflammatory activation, and immune dysregulation leading to impaired placental perfusion, rather than solely by direct infection of placental tissues. We therefore integrate evidence from veterinary research and human studies to examine the biological mechanisms through which leptospirosis may contribute to pregnancy complications and potentially overlap with other pregnancy-related disorders. A One Health approach that combines human, animal, and environmental data is essential to improve disease prevention, reduce its reproductive burden, and protect the health of pregnant individuals and their developing fetuses.

## 1 Introduction

*Leptospira* is a bacterial genus belonging to the phylum Spirochaetota, class Spirochaetes, order Spirochaetales. This order also includes the genera *Treponema* and *Borrelia*, which are widely known as the causative agents of syphilis (*Treponema pallidum*) and Lyme disease (*Borrelia burgdorferi*), respectively.

The genus *Leptospira* comprises both non-pathogenic and pathogenic species. Within the pathogenic group, species range from low-virulence organisms to highly virulent forms that are most commonly associated with human infection (Vincent et al., 2019). Over the past decade, the taxonomy of *Leptospira* has expanded rapidly. In 2018, the description of 12 novel species increased the number of recognized *Leptospira* species to 35 (Thibeaux et al., 2018). Shortly thereafter, comparative genomics analyses further expanded the genus to 64 species (Vincent et al., 2019), revealing a previously underestimated diversity. To date, 74 *Leptospira* species have been reported (Bilung et al., 2026), and this number is expected to continue increasing as advances in sequencing technologies enable the generation of more complete and high-quality bacterial genomes.

Pathogenic *Leptospira* can infect virtually all mammals (Adler and de la Pena Moctezuma, 2010), as well as amphibians (Everard et al., 1988; Gravekamp et al., 1991), reptiles (Paz et al., 2019; Bauso et al., 2020; Rodamilans et al., 2020), and fish (Mgode et al., 2014), demonstrating remarkable evolutionary adaptation to a broad range of hosts. This adaptability is likely driven, at least in part, by traits acquired via horizontal gene transfer, as suggested by features of their genome architecture (Vincent et al., 2019). However, *Leptospira* possess unique virulence factors that complicate our understanding of host-pathogen interactions, as many of these determinants, including atypical lipopolysaccharides (LPS) and surface-exposed adhesins, lack clear counterparts in other well-characterized bacterial species. Adding further complexity, more than 300 *Leptospira* serovars have been described (Adler and de la Pena Moctezuma, 2010; Picardeau, 2017). Serovar classification is based on differences in surface-exposed LPS epitopes and is epidemiologically relevant, as commercially available vaccines are formulated against specific circulating serovars (Barazzone et al., 2021). Moreover, certain host species infected with specific serovars can remain asymptomatic and act as chronic carriers (Ellis, 2015), serving as a major source of animal-to-human transmission.

Human infection results from direct contact with urine from infected animals or indirect exposure through contaminated water or food, and is classically associated with occupational exposure (e.g., farmers, veterinarians) and recreational activities, particularly aquatic sports (Levett, 2001). Densely populated urban areas with poor sanitation also represent high-risk environments, primarily because of the high density of rodents, which serve as the main reservoirs of these bacteria. Globally, human leptospirosis accounts for approximately one million cases annually, although its burden varies substantially across geographical regions (Costa et al., 2015), with tropical countries accounting for an estimated 73% of the global incidence. Seasonal factors further influence disease occurrence, which increases markedly after heavy rainfall and flooding events, such as those recently reported in southern Brazil (Azevedo et al., 2025; Merker Breyer et al., 2025).

*Leptospira* enter the bloodstream through cuts or abrasions in the skin or via intact mucous membranes (Ko et al., 2009). Following systemic entry, leptospirosis is described as a biphasic disease, consisting of an initial acute leptospiremic phase followed by a second immune-mediated phase characterized by the development of a humoral antibody response. After exposure, the incubation period generally ranges from 7 to 12 days, although symptom onset may occur later (Rajapakse et al., 2025). The clinical presentation is variable and depends on multiple factors, including host susceptibility and the infecting serovar. In humans, early symptoms are often non-specific and include fever, headache, vomiting, and fatigue (Bilung et al., 2026), leading to frequent misdiagnosis as influenza-like illness. However, leptospirosis can progress rapidly to severe disease, resulting in renal and hepatic failure, pulmonary hemorrhage that may present with hemoptysis, and death, with reported mortality rates reaching up to 40% (Bilung et al., 2026). Long-term sequelae have been observed in approximately 10% of cases, and clinical follow-up is therefore recommended for several years after recovery (Puliyath and Singh, 2012). Fortunately, despite the presence of antibiotic resistance-associated genes in their genomes, *Leptospira* remain highly sensitive to most commonly used antibiotics (Pineda et al., 2024), including penicillin, which supports effective treatment when therapy is initiated early.

Beyond the systemic clinical manifestations, leptospirosis has also been linked to reproductive disorders. This association is extensively documented in livestock and, to a lesser extent, in humans. In farm animals, reproductive involvement differs from classical leptospirosis, in which the kidneys are the primary target and the main site of leptospiral colonization and replication (Adler and de la Pena Moctezuma, 2010). Instead, reproductive pathology is thought to result from localized infection of the genital tract (Aymee et al., 2024b; Aymee et al., 2024a), which may occur in the absence of systemic symptoms and persist chronically (Aymee et al., 2024a). Adverse outcomes such as infertility, abortion, and agalactia (Ellis, 1994; Aymee et al., 2024b), along with the costs of veterinary care and treatment, contribute significantly to economic losses. Consequently, leptospirosis remains a major concern in countries where livestock production is a primary economic activity.

In contrast to animal infection, reproductive complications in humans are likely to result from systemic dissemination rather than localized genital tract infection. In pregnant individuals, infection has been associated with spontaneous miscarriage, fetal growth restriction, neonatal mortality, and, in severe cases, maternal death (Selvarajah et al., 2021), although the underlying mechanisms remain poorly understood. A deeper understanding of the pathophysiology and epidemiology of leptospirosis during pregnancy is, therefore, needed to support clinical management and public health strategies in regions where leptospirosis is endemic and the risk to pregnant individuals is elevated.

**Figure 1** summarizes the epidemiological cycle of *Leptospira*, as well as the adverse pregnancy and reproductive outcomes associated with leptospiral infection in livestock and humans, which will be further discussed in the following sections.

**Figure 1:**
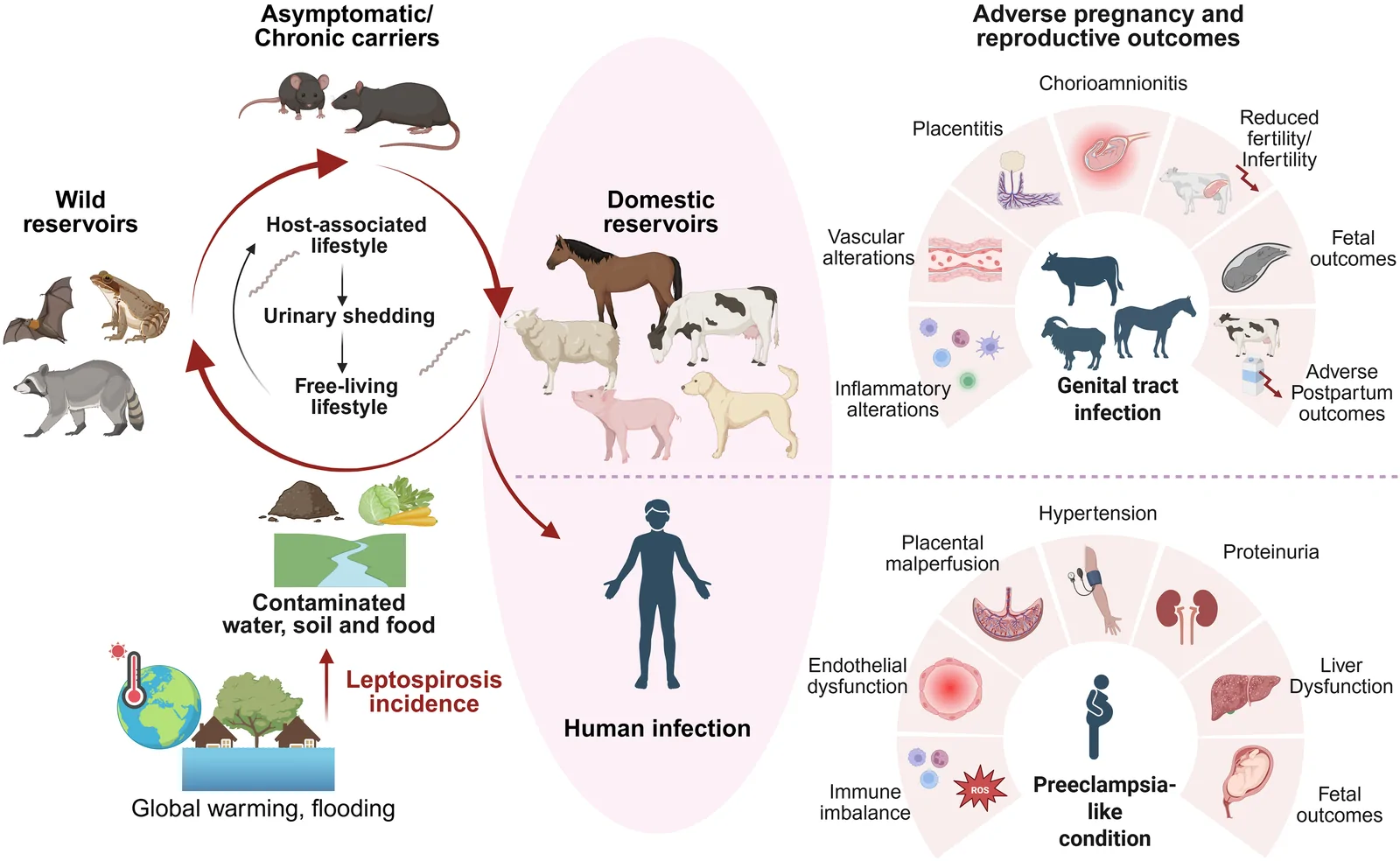
Epidemiological cycle of pathogenic *Leptospira* spp. and associated adverse pregnancy and reproductive outcomes. Most pathogenic *Leptospira* species alternate between host-associated and free-living environmental stages within their epidemiological cycle, enabling efficient transmission across a wide range of hosts. Rodents, in particular, are major chronic carriers and play a central role in leptospirosis transmission (left). Humans are incidental hosts who may become infected as a consequence of exposure to circulating bacteria but do not contribute to sustained transmission. Beyond systemic disease, leptospirosis also affects pregnancy and reproduction, outcomes that are well documented in domestic animals, especially livestock, but remain less characterized in humans, largely due to underreporting and misdiagnosis (right). Clinical overlap with other pregnancy-related disorders, most notably preeclampsia, further contributes to diagnostic challenges. This image was created with BioRender.com.

## 2 Literature search strategy

This narrative review was based primarily on a literature search conducted in PubMed, supplemented by manual screening of the reference lists of relevant articles and reviews. Searches combined the terms “*Leptospira”* or “leptospirosis” with keywords related to pregnancy and reproduction, including “pregnancy”, “placenta”, “fetal”, “abortion”, “stillbirth”, “reproductive failure” and “infertility”. Veterinary and One Health-related searches focused on *Leptospira* included terms such as “cattle”, “bovine”, “equine”, “sheep”, “goat”, “livestock” and “epidemiology”. Literature covering general aspects of *Leptospira* biology, virulence, transmission and host-pathogen interactions was also considered to provide biological or epidemiological context. Additional targeted searches were conducted as needed to explore placental, vascular and immune mechanisms discussed in the review, using terms such as “inflammation”, “immune”, “macrophages”, “endothelial”, “vascular”, “angiogenesis” and “preeclampsia”. Conference proceedings, theses, and reports from authoritative organizations were identified through web searches.

Selected publications included original research articles, case reports and case series, reviews and systematic reviews, and experimental studies related to the mechanisms discussed in the review. Given the limited evidence specifically addressing leptospirosis during pregnancy, no restrictions were applied based on study design or host species. Studies unrelated to the scope of the review were excluded, but articles not specific to *Leptospira* were included selectively when they provided relevant biological context for interpreting findings associated with leptospiral infection and were clearly identified in the text. As this is a narrative rather than a systematic review, no formal risk-of-bias assessment or synthesis of quantitative evidence was performed.

## 3 Reproductive outcomes of leptospiral infection in livestock

Reproductive disorders are among the leading causes of reduced fertility in both large and small ruminants, and leptospirosis represents a significant threat in this context. Clinical outcomes such as abortion, repeat estrus, agalactia, stillbirths, and the birth of weak offspring have been widely reported (Ellis, 1994; Aymee et al., 2024b), contributing substantially to economic losses in livestock production. Although several studies have attempted to quantify these losses in terms of abortion rates and costs associated with preventive measures, most reports are geographically restricted and heavily biased toward cattle. Thus, current projections may fail to capture the true global impact of leptospirosis on animal reproduction (Aymee et al., 2024b). Beyond direct bacterial colonization, accumulating evidence suggests that reproductive pathology may also involve local immune and inflammatory responses within the genital tract and, during pregnancy, inflammatory and vascular alterations at the maternal-fetal interface.

Reproductive tract involvement in leptospirosis has been studied predominantly in females, whereas evidence from several species, including bulls, rams, and boars, also demonstrates male genital tract colonization. In bulls and rams, pathogenic *Leptospira* or leptospiral DNA have been detected in genital tissues and semen, supporting a potential role for males in maintaining and transmitting strains associated with reproductive disease (Soares et al., 2021; Aymee et al., 2024b; Pedrosa et al., 2024). Similarly, *L. santarosai*, also pathogenic, has been detected in the testes and semen of an asymptomatic boar in the absence of histopathological inflammation, suggesting that leptospires can persist within male reproductive tissues without evident tissue damage (Diaz et al., 2022). Moreover, sows artificially inseminated with semen from this boar developed reproductive problems, providing evidence consistent with venereal transmission (Diaz et al., 2022). Experimental studies further support testicular colonization, with viable *L. interrogans* detected in murine testes predominantly during the acute leptospiremic phase, indicating transient rather than chronic colonization (Shetty et al., 2022). Experimental infection of male golden Syrian hamsters with multiple *Leptospira* species and serogroups has likewise demonstrated colonization of several reproductive organs, particularly during acute infection (Silva, 2025). The ability of leptospires to colonize the testis with limited inflammation in some hosts, together with the immune-privileged environment of this organ (Fijak and Meinhardt, 2006), suggests that the testis may provide a protected niche that favors bacterial survival and potentially facilitates venereal transmission. Evidence for sexual transmission in humans remains limited (Stollberg et al., 2026).

### 3.1 Cattle

In cattle, *Leptospira* spp. have been detected in multiple tissues and secretions of the female reproductive system, including the ovaries and ovarian follicles, the oviducts (Dos Santos Pereira et al., 2022), the uterus (Aymee et al., 2022), and vaginal fluid (Pimenta et al., 2019). *Leptospira* has also been detected in the male reproductive tract (Aymee et al., 2024b), although it remains comparatively less studied. Reproductive failure in cattle has been associated with both host-adapted and incidental strains, although the degree of host-strain adaptation may influence infection persistence and clinical outcomes. Differences in tissue tropism, persistence, and host inflammatory responses may therefore shape disease manifestations. Indeed, abortion and fertility disorders are generally, though not exclusively, associated with chronic infection (Sohm et al., 2023), which may involve persistent colonization and localized immune activation within reproductive tissues.

Reproductive losses have been reported throughout gestation, from early embryonic death to late-term abortion (Aymee et al., 2024b). Studies conducted in endemic areas have identified an association between seropositivity and infertility, with infertility risk increasing alongside urinary leptospiral shedding (Macchi et al., 2024). However, other studies have shown poor concordance between serological evidence of exposure and genital colonization. In some cohorts, leptospiral DNA was detected in reproductive tissues in only 6% of animals, despite seroprevalence rates reaching 20% within the same group (Vidal et al., 2017). These findings highlight important gaps in understanding the sequence of events leading to reproductive failure, including whether placental infection follows hematogenous dissemination, arises from pre-existing genital colonization, or whether both routes can occur, and how localized immune responses contribute to reproductive injury.

Transplacental transmission of *Leptospira* is well established, as bacteria have been detected in aborted bovine fetuses (Ellis, 1994; Aymee et al., 2023). Reported fetal findings include jaundice and hemorrhagic lesions, while anicteric fetuses have also been described. Additional reported lesions include enlarged livers with focal necrosis and signs of hypoxic injury, such as petechial hemorrhages in multiple organs, including the thymus, thyroid, lungs, heart, and surfaces such as the pleura, peritoneum, and mesentery (Ellis, 1994; Aymee et al., 2023). In the kidneys, tubular necrosis with immune cell infiltration may reflect a combination of direct bacterial and inflammation-mediated injury. Notably, abortions have also been reported in the absence of obvious fetal lesions, raising the possibility that placental dysfunction may contribute to pregnancy loss (Aymee et al., 2023), although the mechanisms remain poorly characterized. Consistently, cows from herds experiencing abortions and fertility issues were negative for leptospiral shedding in urine but positive in vaginal fluid, with identical serotypes identified in dams and aborted fetuses (Aymee et al., 2023). Together, these observations reinforce the relevance of genital tract colonization as an important extra-renal site of leptospiral persistence, with potential consequences for reproductive outcomes.

### 3.2 Equines

Genital colonization by *Leptospira* has also been reported in equines, including animals lacking systemic clinical signs (Aymee et al., 2024a), supporting the genital tract as an additional site of leptospiral persistence. Nonetheless, systemic dissemination remains a plausible route of uterine infection, particularly during bacteremia. In fact, leptospiral placentitis in mares is generally considered a diffuse or hematogenous form of placentitis, in which the bacteria reach the uterus through systemic circulation (Canisso, 2015). In this scenario, systemic inflammation, along with hyperthermia and increased prostaglandin production, may contribute to the pregnancy loss (Canisso, 2015) by disrupting placental function and uterine homeostasis.

Placental abnormalities are frequently observed and include edema, hemorrhage, and placental detachment (Aymee et al., 2024a). Histopathological findings such as necrotizing placentitis and vasculitis indicate a robust local inflammatory response, likely impairing nutrient and oxygen exchange at the maternal-fetal interface. As observed in cattle, genital leptospiral infection in horses has been associated with abortion, infertility, and the birth of weak foals (Aymee et al., 2024a). In some breeds, placentitis is estimated to affect up to 5% of pregnancies (Canisso, 2015), and *Leptospira* has been reported as the second most frequently identified pathogen in cases of equine placentitis, after *Streptococcus equi* (15.7% vs 16.5%, respectively) (Canisso, 2015).

Proteomic analyses of amniotic and allantoic fluids in mares using a non-leptospiral model of experimentally induced placentitis have revealed significant molecular alterations consistent with placental insufficiency and fetal stress. Proteins involved in inflammation, coagulation, and molecular transport, including haptoglobin, antithrombin III, alpha-1-antiproteinase 2, alpha-2-HS-glycoprotein, and transferrin, were upregulated (Loux and Ball, 2018), suggesting inflammatory and hemostatic alterations that may affect placental perfusion. In contrast, structural proteins such as collagen alpha-1(I) chain and several cytoskeletal keratins were downregulated (Loux and Ball, 2018), indicating compromised tissue integrity. Notably, the upregulation of serine protease inhibitors, including alpha-1-antitrypsin (SERPINA1) and serpin A3-8 (SERPINA3-8), in fetal fluids (Loux and Ball, 2018), mirrors molecular signatures observed in preeclampsia (PE) in humans, where these factors are elevated either at the mRNA or protein level in placental tissues or maternal urine, respectively (Chelbi et al., 2007; Chelbi et al., 2012; Buhimschi et al., 2014). Although these molecular alterations have not been demonstrated specifically in leptospiral placentitis, *Leptospira-*specific pathological studies reveal inflammatory and vascular injury at the maternal-fetal interface, suggesting that similar pathogenic processes may occur during leptospiral infection.

Consistent with this possibility, *Leptospira* has been detected in multiple organs of aborted equine fetuses, including the kidneys, liver and lungs (Canisso, 2015). Lesions have been reported in up to 80% of examined cases, affecting both fetuses and placentas (Poonacha et al., 1993). In fetuses, lesions have been described particularly in the kidneys and liver, including swelling and edema (Poonacha et al., 1993). At the maternal-fetal interface, lesions have been reported in the allantochorion, amnion and umbilical cord. Umbilical cord lesions include edema, hemorrhage, inflammatory cell infiltration, and, in cases of leptospiral funisitis, an exudate containing neutrophils and fibrin (Poonacha et al., 1993; Sebastian et al., 2005), indicative of severe localized inflammation. Placental histopathology has further revealed leukocytic infiltration, stromal edema, villous atrophy and necrosis, vasculitis, and vascular thrombosis (Poonacha et al., 1993; Donahue and Williams, 2000). The occurrence of placental inflammation together with leptospiral funisitis is particularly relevant, as acute chorioamnionitis and funisitis are commonly associated with ascending microbial infections from the lower genital tract (Kim et al., 2015). These observations suggest that, in addition to hematogenous dissemination, an ascending route of infection accompanied by local immune-mediated inflammation may contribute to pregnancy loss.

### 3.3 Small ruminants

Small ruminants, including sheep and goats, are generally considered less susceptible to leptospirosis. Nevertheless, reproductive failure associated with leptospiral infection has been well documented in these species. Reported outcomes include abortion, most commonly in late gestation, stillbirth, and the birth of weak offspring (Ellis, 1994; Dorsch et al., 2021). Infection in small ruminants is frequently linked to strains more commonly circulating in cattle (Martins and Lilenbaum, 2014), underscoring the relevance of interspecies transmission and the remarkable adaptability of *Leptospira* to persist across diverse hosts with distinct immune landscapes.

As in other animal species, fetuses from infected small ruminants are frequently affected, with leptospires detected primarily in the liver and kidneys. Some reports indicate that up to 70% of aborted fetuses show severe maceration with advanced autolysis and tissue degradation (Martins et al., 2012; Dorsch et al., 2021), consistent with intrauterine death occurring prior to abortion. This supports the hypothesis that persistent maternal systemic infection and associated inflammatory responses may progressively compromise placental function, with pregnancy loss resulting from placental dysfunction rather than solely from acute fetal leptospiral septicemia.

## 4 Immune and vascular involvement in human leptospirosis during pregnancy

In contrast to animal leptospirosis, comparatively fewer studies have investigated reproductive outcomes in humans. Most available evidence derives from case reports and small observational studies (Selvarajah et al., 2021), with a notable lack of large-scale research assessing incidence, particularly in high-risk areas, such as endemic or flood-prone regions. Moreover, surveillance systems in many low-income countries remain limited, further contributing to the underestimation of the disease burden. In addition, regional variability in exposure risk complicates the generation of accurate global estimates (Picardeau, 2015). As a result, leptospirosis remains an underappreciated cause of maternal morbidity and adverse pregnancy outcomes, despite the causative pathogen having been known for more than a century. Notably, the first systematic review on leptospirosis in pregnancy was only published in 2021 (Selvarajah et al., 2021).

The incidence of leptospirosis during pregnancy has been estimated at 1.3 per 10,000 individuals presenting with fever or jaundice (Selvarajah et al., 2021). However, this estimate does not capture the full spectrum of clinical manifestations and is based on studies that were largely rated as fair to poor in quality (Selvarajah et al., 2021). The geographic distribution of these studies is uneven, with nearly half originating from Europe. In regions where leptospirosis is endemic and strongly influenced by flooding and other natural disasters, including much of Latin America, limited reporting continues to hinder accurate estimation of incidence. Indeed, reports from this region account for only around 16% of published studies (Selvarajah et al., 2021).

Among pregnant individuals ultimately diagnosed with leptospirosis, commonly reported symptoms include fever, jaundice, vomiting, and myalgia (Coghlan and Bain, 1969; Shaked et al., 1993; Chedraui and San Miguel, 2003; Gaspari et al., 2007; Gainder et al., 2010; Hicham et al., 2013; Koe et al., 2014; Tong and Mathur, 2018; Patel et al., 2023). Although conjunctival suffusion is classically described as a characteristic feature of leptospirosis (Puliyath and Singh, 2012), it is absent in most reported pregnancy-associated cases (Selvarajah et al., 2021). In practice, clinical suspicion of leptospirosis was frequently prompted by epidemiological factors rather than by clinical signs, particularly contact with farm animals (Coghlan and Bain, 1969; Gainder et al., 2010; Sutter et al., 2023). One illustrative case described intrauterine fetal death with fetal maceration in a woman living in close contact with pigs and dogs that tested positive for *Leptospira* (Coghlan and Bain, 1969), reinforcing the relevance of household and environmental exposure in shaping pregnancy risk. Severe complications such as disseminated intravascular coagulopathy and sickle cell crisis (Selvarajah et al., 2021) have also been reported in pregnant patients with leptospirosis. In the context of sickle cell disease, intravascular hemolysis leads to the release of damage-associated molecular patterns (DAMPs) into the circulation, which can trigger inflammatory pathways that promote endothelial activation and injury, thereby contributing to an increased risk of pregnancy-related vascular complications such as PE (Prophet et al., 2018).

Despite the presence of placental abnormalities, pregnancy complications associated with leptospirosis appear in most cases to result from systemic bacteremia and secondary placental involvement (Shaked et al., 1993; Koe et al., 2014; Tong and Mathur, 2018; Sutter et al., 2023), rather than from primary genital tract infection, which has not been reported to date. Once leptospires proliferate sufficiently in blood and tissues, bacterial proteins and their interactions with host components may contribute to the onset of clinical manifestations. Recent studies using recombinant proteins and interaction assays have further characterized these mechanisms. For example, the leucine-rich repeat protein LIC11505 binds host collagen type I, fibronectin, glycosaminoglycans and several integrin receptors, supporting a role in host tissue attachment (Foltran et al., 2024). Similarly, LIC12254 interacts with human αVβ8 and α8 integrins through its RGD motif (Cavenague et al., 2023), which could potentially facilitate bacterial internalization through integrin activation (Geiger et al., 2009). *In silico* analyses and *in vitro* studies have identified additional surface-associated proteins, such as LIC11098 (Gaspar et al., 2024), LBL2618 (Kamaruzaman et al., 2024), and LIC10271 (Sarma et al., 2023), that interact with several host extracellular matrix (ECM) components and plasma proteins, revealing a broad repertoire of leptospiral proteins capable of interacting with the host. Other leptospiral proteins directly interact with host cell adhesion molecules. LIC13411 binds VE-cadherin and facilitates leptospiral association with endothelial cells and host tissues (Surdel et al., 2022). Additional leptospiral adhesins also target cadherins. LIC11711 and LIC12587 bind E-cadherin (Kochi et al., 2019), whereas LIC10831 binds both E-cadherin and VE-cadherin (Eshghi et al., 2019). In addition, the secreted metalloprotease leptolysin degrades extracellular matrix components and induces hemorrhagic lesions *in vivo*, providing a direct link to vascular injury (Courrol et al., 2022). Indeed, a central pathological feature of leptospirosis is endothelial damage affecting small vessels and leading to ischemia across multiple organs (Adler and de la Pena Moctezuma, 2010). Many manifestations observed in pregnancy, including renal tubular necrosis, hepatocellular injury, and placentitis, are consistent with widespread endothelial dysfunction. Although immune responses and antibiotic therapy can eliminate bacteria, long-lasting tissue damage, such as myocarditis, may persist (Adler and de la Pena Moctezuma, 2010).

At the molecular level, endothelial injury in leptospirosis reflects a complex interplay between leptospiral virulence factors and host immune responses. *Leptospira-*associated molecules, including structurally atypical LPS, are preferentially recognized in humans by toll-like receptor 2 (TLR2) rather than TLR4, which mediates classical LPS responses (Rajapakse et al., 2025). This recognition triggers activation of MAPK- and NF-κB-dependent signaling pathways, resulting in robust production of pro-inflammatory cytokines such as TNF-α, IL-1β, IL-6, and IL-8, as well as reactive oxygen and nitrogen species, which serve as antimicrobial defenses. Concomitantly, increased expression of anti-inflammatory mediators, including TGF-β, IL-10, IL-13, and IL-4, has been reported, contributing to immune dysregulation characterized by a systemic cytokine storm, immunoparesis, and sustained inflammation (Haake and Levett, 2015; Rajapakse et al., 2025). A mechanistic link between dysregulated host immunity, vascular injury and tissue pathology during *Leptospira* infection is supported by experimental animal models. A comparative study using resistant OF1 mice and susceptible golden Syrian hamsters infected with virulent *Leptospira interrogans* showed markedly different inflammatory responses (Matsui et al., 2011). Mice exhibited an early but transient induction of the pro-inflammatory mediators IL-1β, IL-6, TNF-α, CCL3 and CXCL10, together with earlier induction of the anti-inflammatory cytokine IL-10 in blood and target tissues, whereas hamsters showed delayed and sustained overexpression of these mediators, accompanied by edema, vascular congestion, hemorrhage and inflammatory infiltration in multiple organs (Matsui et al., 2011). Since CCL3 and CXCL10 promote leukocyte recruitment and trans-endothelial migration, and IL-1β and TNF-α enhance leukocyte adhesion to endothelial cells by inducing adhesion molecules (Matsui et al., 2011), their sustained and pronounced overexpression in susceptible hamsters provides a plausible mechanism linking systemic inflammation to endothelial activation and vascular injury. Consistently, increased expression of IL-1β, CCL3 and CXCL10 has also been associated with severe disease in another lethal hamster model, in which pulmonary hemorrhage and vascular congestion were among the earliest pathological alterations and preceded liver and kidney injury (Philip et al., 2022). By contrast, a sublethal mouse model of infection has been shown to produce a predominantly organ-specific inflammatory response, with renal upregulation of TNF-α, IL-1β and IL-6 and leukocyte-recruiting chemokines, among others, accompanied by mononuclear infiltration, tubular damage and altered glomerular structure (Richer et al., 2015). Altogether, these studies suggest that the kinetics, magnitude and tissue distribution of the host inflammatory response are closely associated with the severity and pattern of vascular and organ injury during leptospirosis.

Specific host-pathogen interactions may also shape immune activation and disease progression, as evidenced by comparative studies of pathogenic and saprophytic *Leptospira*. In a susceptible mouse model, non-pathogenic *L. biflexa* induced an early chemokine response without detectable cytokine production, involving macrophages, dendritic cells, neutrophils and NK cells, sufficient to promote bacterial clearance and prevent systemic dissemination. In contrast, pathogenic *L. interrogans* triggered a much broader pro- and anti-inflammatory cytokine and chemokine response, involving T cells in addition to innate cells. Despite a more robust immune activation, *L. interrogans* disseminated into the bloodstream (Shetty et al., 2021), which may reflect immune evasion mechanisms associated with pathogenic leptospires. The involvement of adaptive immunity during persistent *L. interrogans* infection has also been demonstrated in C57BL/6J-based mouse models, including wild-type and TLR4-deficient animals. Splenic CD4^+^ T cells showed increased differentiation toward an effector phenotype, whereas CD8^+^ effector T cell responses were more prominent under conditions of impaired TLR4 signaling (Nair et al., 2020). Pathogenic and saprophytic leptospires also interact differently with the endothelium. Exposure of human endothelial cells to *L. interrogans* resulted in transcriptional changes affecting pathways involved in actin organization, focal adhesion and ECM interactions, consistent with reduced cellular adhesion to the ECM and cytoskeletal remodeling. These changes were accompanied by disruption of endothelial monolayer integrity (Martinez-Lopez et al., 2010), which may facilitate bacterial passage across endothelial barriers. Conversely, *L. biflexa* induced largely opposite transcriptional changes and caused little disruption of the cellular morphology or monolayer integrity (Martinez-Lopez et al., 2010). Moreover, highly virulent leptospires triggered stronger endothelial inflammatory responses when compared with low-virulence and saprophytic strains, together with greater survival in the bloodstream and increased kidney and bladder tissue burdens *in vivo* (Anderson et al., 2026). Notably, five of the six highly virulent strains evaluated stimulated significantly higher endothelial production of CCL2, a chemokine that promotes monocyte and macrophage recruitment, potentially contributing to local inflammation and endothelial injury. These strains also displayed greater association with endothelial cells and more disruption of VE-cadherin organization (Anderson et al., 2026).

Beyond these differential endothelial responses, pathogenic *Leptospira* can further compromise vascular integrity through direct interactions with host plasma proteins and modulation of hemostasis. Several leptospiral surface proteins interact with components of the coagulation and fibrinolytic systems. The adhesins LigA and LigB bind fibrinogen, while the outer membrane protein OmpL1, the endostatin-like protein LenA, and the membrane protein MPL36 interact with plasminogen (Choy et al., 2007; Verma et al., 2010; Fernandes et al., 2012; Daroz et al., 2021; Zhu et al., 2023; Rajapakse et al., 2025). Among these proteins, LigB has particularly strong functional evidence supporting a role in hemostatic disruption. It not only binds fibrinogen and factor XIII but can also inhibit fibrin clot formation, as well as platelet adhesion and aggregation (Lin et al., 2011; Hsieh et al., 2016). LIC13086 also binds fibrinogen and plasminogen, inhibiting thrombin-induced fibrin formation, while bound plasminogen can be converted to active plasmin, further promoting fibrinolysis and tissue degradation and thereby facilitating bacterial dissemination (Passalia et al., 2021). Consistent with these mechanisms of vascular damage, experimental and clinical studies have demonstrated disruption of adherens junction proteins, damage to the endothelial glycocalyx, and elevated circulating markers of endothelial activation and injury, such as soluble E-selectin and von Willebrand factor (vWF), which correlate with disease severity (Kornacki et al., 2021; Wickramasinghe et al., 2025). Notably, these markers are also elevated in PE and are widely used as indicators of endothelial and placental dysfunction (Kornacki et al., 2021). Furthermore, CD34, a transmembrane glycoprotein expressed by endothelial cells and involved in vascular homeostasis and leukocyte trafficking, has also been implicated in leptospiral vascular pathology. Reduced CD34 expression in pulmonary capillary walls has been reported in severe leptospirosis, supporting the presence of systemic microvascular injury (De Brito et al., 2018). Its downregulation has been linked to PE (Liu et al., 2015) and is strongly associated with pyroptosis in placental endothelial cells (Feng et al., 2025). These observations suggest a potential mechanistic intersection between infection-induced endothelial injury and pregnancy-related vascular pathology.

Consistent with the endothelial and immune alterations described above, leptospirosis in pregnancy shares clinical and pathological features with other pregnancy-related disorders, including Haemolysis, Elevated Liver Enzymes and Low Platelets (HELLP) syndrome and PE (Selvarajah et al., 2021), contributing to diagnostic overlap and under-recognition. This overlap also extends to platelet abnormalities. Thrombocytopenia is common in severe leptospirosis and is associated with hemorrhagic complications and severe clinical outcomes (Turgut et al., 2002; Daher et al., 2014), although its underlying mechanisms remain incompletely understood. Virulent *L. interrogans* can directly interact with human platelets *in vitro*, inducing platelet detachment, membrane disruption, and fragmentation without directly triggering platelet activation or aggregation (Vieira and Nascimento, 2020). In addition, as discussed above, LigB can interfere with fibrinogen-dependent platelet adhesion and aggregation, while interactions between platelets and activated leukocytes, specifically neutrophils, may further contribute to platelet consumption and thrombocytopenia (Lin et al., 2011; Santecchia et al., 2020). Clinical studies further indicate that bleeding complications are associated with platelet dysfunction in addition to thrombocytopenia, with increased vWF-platelet binding potentially contributing to platelet activation and clearance (Tunjungputri et al., 2017). This is particularly relevant to HELLP syndrome, in which endothelial injury promotes platelet adhesion, activation, and consumption within the microvasculature, contributing to thrombocytopenia and thrombotic microangiopathy (Haram et al., 2009; Abildgaard and Heimdal, 2013). Endothelial dysfunction is central to the pathophysiology of PE (Boeldt and Bird, 2017). Notably, hypertension and proteinuria, hallmark features of PE, were reported in 8% and 48% of leptospirosis cases, respectively (Selvarajah et al., 2021). Additionally, 56% of cases presented cardiovascular abnormalities (Selvarajah et al., 2021), a percentage that is likely underestimated, as many studies did not systematically evaluate cardiovascular involvement (Selvarajah et al., 2021). Importantly, PE is characterized by an immune imbalance skewed toward a pro-inflammatory state in both maternal circulation and the placenta (Cornelius, 2018), contributing to sustained endothelial activation and progressive placental injury. Platelet activation is also increased in PE and can participate in placental thrombo-inflammatory responses (Kohli et al., 2016; Singh et al., 2025), which could likewise be promoted during leptospirosis through platelet dysfunction and endothelial activation. In this context, maternal infection with diverse pathogens, including *Leptospira*, may further potentiate inflammatory signaling (Nieves et al., 2024), thereby exacerbating endothelial dysfunction and amplifying placental damage.

Another important consideration is that maternal immune system undergoes dynamic adaptations throughout pregnancy to maintain fetal tolerance while preserving antimicrobial defense. These changes affect both the composition of immune cell populations and their responses to specific stimuli. Mass cytometry analysis has shown cell-specific adaptations, including increased neutrophil abundance and reactivity, enhanced IFN-α responses in NK cells and monocytes, modulation of TLR4 signaling in myeloid dendritic cells, and progressive changes in signaling pathways involving T cells (Aghaeepour et al., 2017). Importantly, not all immune cells are affected in the same way, and immune modulation varies across gestational stages. For example, TLR4 responses to LPS were reduced in myeloid dendritic cells early in pregnancy, whereas neutrophil responsiveness to LPS progressively increased across gestation (Aghaeepour et al., 2017). Similarly, TLR4-induced TNF-α production by monocytes was enhanced in the first trimester but decreased later in pregnancy (Ziegler et al., 2018). As discussed for resistant and susceptible animal models of *Leptospira* infection, disease severity appears to depend, among other factors, on the timing and regulation of the immune response (Shetty et al., 2021). It is well established that severe leptospirosis is associated with increases in circulating cytokines, including IL-1β, IL-6, IL-8, TNF-α, and IL-10, with IL-6 and IL-10 independently associated with fatal outcomes (Reis et al., 2013). Moreover, this generalized cytokine dysregulation may promote sustained endothelial permeabilization, facilitating systemic bacterial dissemination and contributing to progression toward a sepsis-like syndrome (Cagliero et al., 2018). In this context, immune and metabolic alterations during pregnancy, including expansion of myeloid populations, relative reductions in lymphocyte populations, and, particularly in late pregnancy, increased cortisol and reduced insulin sensitivity, partially mirror features observed in sepsis and may lower the threshold for developing a maladapted host response to infection (Sharma et al., 2022). Conversely, if infection progresses to sepsis, a rapid inflammatory cascade characterized by increased TNF-α, IL-6, and IL-1β can promote microvascular thrombosis, increased vascular permeability, and capillary leak. These alterations may contribute to maternal vascular malperfusion and placental hypoxia, compromising pregnancy progression (Amarasinghe et al., 2026). Indeed, maternal sepsis has been associated with adverse pregnancy outcomes, including preterm birth, fetal growth restriction, and stillbirth (Amarasinghe et al., 2026).

Placental abnormalities associated with human leptospirosis include ischemia and focal abruption (Koe et al., 2014), subtotal infarction and fibrin deposition (Sutter et al., 2023), as well as perivillous fibrinoid accumulation and intervillositis (Tong and Mathur, 2018). Together, these alterations suggest significant impairment of placental perfusion accompanied by inflammatory injury at the maternal-fetal interface. Massive perivillous fibrin deposition and intervillositis are characterized by infiltration of maternal immune cells into the placenta and disruption of the intervillous space (Cornish et al., 2022), further compromising placental perfusion. Among the infiltrating immune populations, maternal macrophages, CD4^+^ and CD8^+^ T lymphocytes are commonly observed, while activation of Hofbauer cells, the resident fetal macrophages of the placenta (Cornish et al., 2022), may also occur. In the context of *Leptospira* infection, placental exposure to bacterial antigens may trigger localized maternal immune activation, promoting recruitment of these maternal immune cells. CD4^+^ T cells are likely to contribute by coordinating inflammatory responses and macrophage activation, whereas CD8^+^ T cells may exacerbate placental tissue injury through cytotoxic and pro-inflammatory mechanisms. The involvement of macrophages, CD4^+^ and CD8^+^ T cells in the immune response to leptospirosis has been demonstrated previously (Guo et al., 2010; Xue et al., 2013; Volz et al., 2015). Activation of Hofbauer cells may also play a role in the placental response to *Leptospira.* Beyond their established functions in angiogenesis, vasculogenesis, and tissue remodeling, Hofbauer cells contribute to fetal immune protection during pregnancy (Pantazi et al., 2022). Inflammatory mediators and tissue damage within the chorionic villi may promote their activation, which, once initiated, could amplify local inflammation and contribute to villous dysfunction. Given that vertical transmission to the fetus has been reported (Shaked et al., 1993; Koe et al., 2014), it is plausible that *Leptospira* reaches the placenta, breaches the placental barrier, and interacts directly with fetal macrophages, as previously hypothesized (Nieves et al., 2024). TLR2-mediated recognition of leptospiral components could also contribute to Hofbauer cell activation (Werts et al., 2001), while bacterial internalization may occur through endocytic mechanisms (Zhao et al., 2022), although neither process has been demonstrated in placental tissue. Of note, *Leptospira* has been shown to enter and subsequently exit human macrophages without evidence of intracellular replication (Santecchia et al., 2022). Macrophage-derived reactive oxygen species (ROS) and nitric oxide can contribute to leptospiral killing (Chen et al., 2023). However, pathogenic *Leptospira* also possess mechanisms that may promote resistance to oxidative killing. Highly virulent *Leptospira* species exhibit increased expression of the catalase-encoding gene *katE*, higher catalase activity, and greater tolerance to peroxide, reflecting an adaptation to oxidative stress (Giraud-Gatineau et al., 2024). Consistently, KatE has been shown to be required for resistance to H_2_O_2_ and to contribute to leptospiral virulence (Eshghi et al., 2012). These mechanisms could favor transient bacterial survival following macrophage internalization, although whether they operate in Hofbauer cells remains unknown. Such interactions could represent a plausible mechanism of fetal exposure.

The major host-pathogen interactions described above, together with their consequences for the three main pathological axes, namely inflammation, endothelial damage, and placental dysfunction, are outlined in **Figure 2**. These mechanisms translate clinically into a spectrum of fetal outcomes. Most pregnant individuals described in published reports were in late pregnancy, and abortion was the most frequently observed event (Shaked et al., 1993; Cardenas-Marrufo et al., 2016). Cases of intrauterine fetal death (Coghlan and Bain, 1969; Tramoni et al., 2003; Gainder et al., 2010) have also been documented, including instances of fetal maceration (Coghlan and Bain, 1969). Finally, although vertical transmission to the fetus or neonate has been demonstrated (Shaked et al., 1993; Koe et al., 2014), many reported cases resulted in the birth of apparently healthy neonates (Chedraui and San Miguel, 2003; Gaspari et al., 2007; Hicham et al., 2013; Tong and Mathur, 2018; Patel et al., 2023), suggesting that additional host, placental, and infection-related factors modulate disease severity.

**Figure 2:**
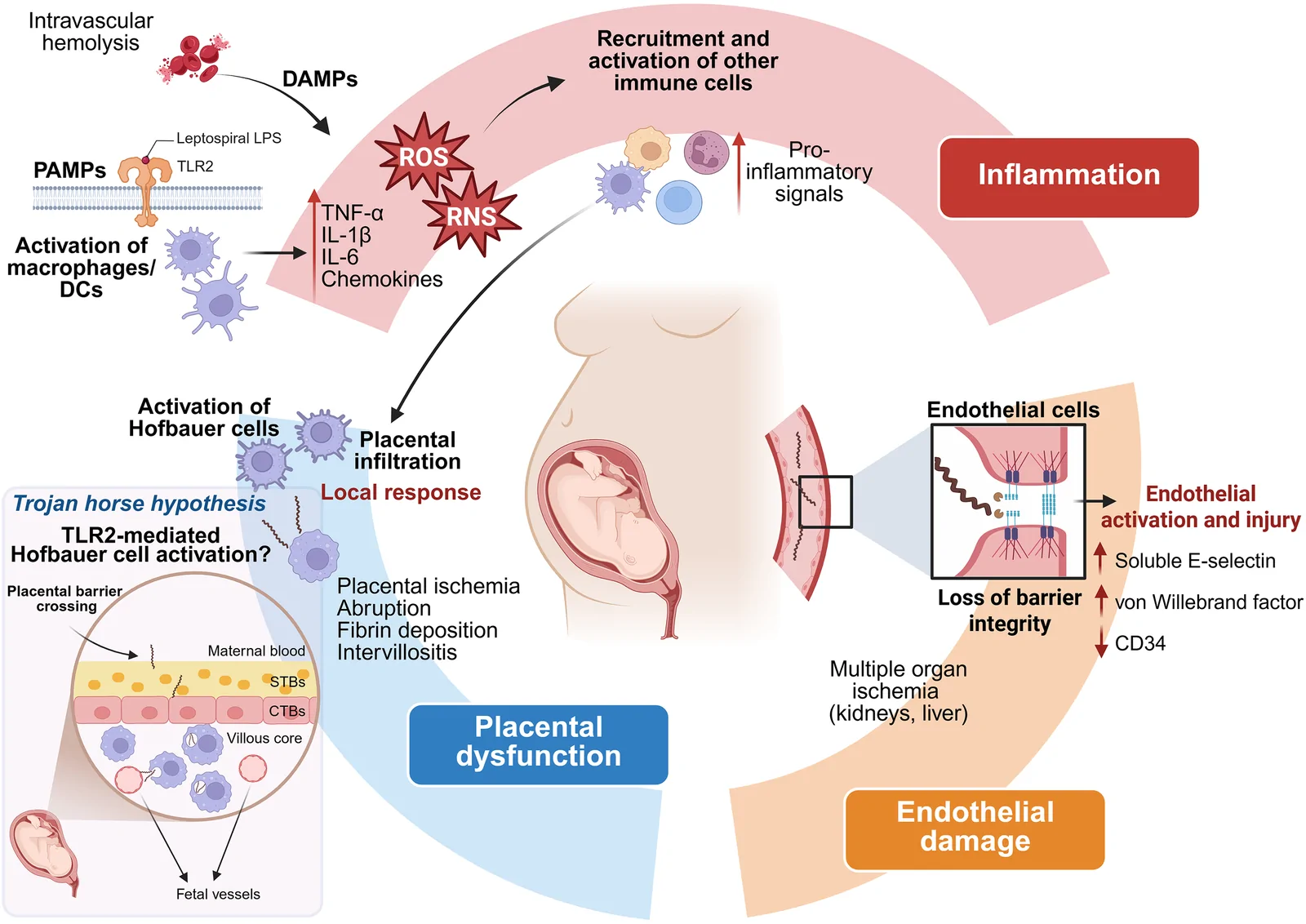
Interplay between inflammation, endothelial injury, and placental dysfunction in human leptospirosis during pregnancy. Recognition of pathogen-associated molecular patterns (PAMPs) and damage-associated molecular patterns (DAMPs) by innate immune cells induces immune activation and the production of pro-inflammatory cytokines, chemokines, and oxidative mediators, including reactive oxygen and nitrogen species (ROS and RNS). This inflammatory milieu promotes recruitment and activation of additional immune populations, including T cells, amplifying systemic inflammation and contributing to endothelial activation and injury. In parallel, *Leptospira* membrane proteins and adhesins disrupt adherens junctions, enabling bacterial dissemination across endothelial barriers and resulting in vascular dysfunction and tissue ischemia. Although the mechanisms of placental involvement remain unclear, inflammatory infiltration at the maternal-fetal interface may activate local immune responses, including Hofbauer cells, contributing to placental injury. In addition, bacterial extracellular matrix-degrading factors and motility-associated mechanisms may facilitate transplacental passage across syncytiotrophoblast (STB) and cytotrophoblast (CTB) layers (Nieves et al., 2024). Once within the villous core, *Leptospira* may interact with or be internalized by Hofbauer cells, where transient survival could potentially facilitate bacterial persistence and dissemination toward fetal vessels, consistent with a proposed “Trojan horse” mechanism. Although this has not been demonstrated in placental tissue, it could provide a plausible explanation for documented fetal exposure and infection. This image was created with BioRender.com.

## 5 Preventive and protective measures

Prevention of leptospirosis relies primarily on reducing exposure to infected animals and contaminated environments, particularly in endemic and flood-prone regions. Individuals should avoid contact with floodwater, sewage, and potentially contaminated soil or freshwater whenever possible, and use waterproof footwear, gloves, and protective clothing when exposure cannot be avoided. Covering skin lesions and ensuring access to safe drinking water are particularly important following flooding or heavy rainfall. Awareness of recent environmental or occupational exposure should also be communicated to healthcare providers to facilitate early recognition and treatment, particularly during pregnancy, given the potential maternal and fetal consequences of delayed diagnosis (Centers for Disease Control and Prevention, 2026).

At the community level, local and national authorities can play an important role through improved sanitation, drainage, waste management, and access to safe water, thereby reducing environmental contamination with leptospires. Rodent control is also important, as rodents are major reservoirs and can continuously contaminate soil, water, and household environments through urinary shedding. Measures such as reducing access to food and waste, limiting rodent infestation, and improving general hygiene can therefore complement individual protection. Animal health interventions are also relevant. Vaccination of livestock and companion animals can reduce clinical disease and may reduce transmission risk. However, protection is largely serovar-specific, and vaccine effectiveness depends on alignment with locally circulating serovars. Moreover, animal vaccination does not always prevent renal carriage, further supporting the need to combine vaccination with reservoir and environmental control (World Health Organization, 2003; Antoniolli et al., 2025). Human vaccines are licensed in only a limited number of countries and provide restricted serovar- or serogroup-specific protection. Their use during pregnancy remains poorly characterized, and pregnancy-specific safety and efficacy data are insufficient to support routine vaccination of pregnant individuals (Rajapakse et al., 2025).

Because leptospirosis transmission occurs at the interface between humans, animal reservoirs, and contaminated environments, these preventive measures should be integrated within a One Health framework. Coordinated surveillance of human and animal infection, characterization of locally circulating strains, environmental monitoring, animal vaccination, public awareness, and targeted interventions following floods or outbreaks can improve identification of transmission sources and guide locally appropriate prevention strategies. Recent One Health analyses have demonstrated positive associations between *Leptospira* exposure in humans, livestock, and environmental compartments, further supporting integrated approaches to disease prevention (Antoniolli et al., 2025).

## 6 Discussion and future directions

The epidemiology of leptospirosis is highly complex, involving both host-associated stages and free-living environmental phases within its transmission cycle. *Leptospira* thrives in tropical and subtropical regions, where temperature and humidity conditions favor bacterial survival and transmission. Climate change has expanded these favorable environmental niches, contributing to the geographic spread of leptospirosis. In parallel, global warming has increased the frequency and severity of natural disasters, such as flooding, which disproportionately affect socioeconomically disadvantaged populations lacking adequate infrastructure. Because *Leptospira* can persist in water and mud, flooding events create optimal conditions for widespread exposure of both animal and human hosts. Among recognized risk groups, pregnant individuals represent a particularly vulnerable population due to the potential consequences for both maternal and fetal health.

Clinical and histopathological findings indicate that adverse pregnancy outcomes associated with human leptospirosis appear to be more closely linked to systemic endothelial dysfunction and consequent placental malperfusion, as evidenced by placental ischemia and increased intervillous fibrin deposition (Koe et al., 2014; Sutter et al., 2023), than to primary placental infection. Nevertheless, when *Leptospira* reaches the placenta, direct interactions with placental cell receptors may trigger additional pathogenic responses, with inflammation acting as a central facilitator (Nieves et al., 2024). Maternal inflammation may further exacerbate placental damage by promoting intervillositis, fibrinoid necrosis, and oxidative stress, ultimately impairing maternal-fetal exchange and creating a hypoxic intrauterine environment, as described in PE (Cornelius, 2018). Thus, leptospiral infection may converge on shared downstream pathways involving angiogenic imbalance, coagulation disturbances, and inflammation-driven endothelial injury, potentially resulting in PE-like clinical and pathological features. Although leptospiral placental crossing has been documented (Shaked et al., 1993; Koe et al., 2014), the available evidence suggests that fetal injury may frequently result from inflammation-associated vasculopathy and placental hypoxia.

Several important knowledge gaps remain regarding the pathogenesis and clinical consequences of leptospirosis during pregnancy. Mechanistic studies are needed to determine how *Leptospira* reaches and colonizes placental tissues and to identify the bacterial and host factors that determine maternal-fetal transmission and pregnancy outcomes. *In vitro* models of the placental interface, including 2D cultures, organoids, and microfluidic placenta-on-a-chip platforms, provide promising systems to investigate interactions of leptospires and their surface proteins with trophoblasts, endothelial cells, and ECM components and to determine whether these interactions facilitate placental colonization and transplacental dissemination (Cherubini et al., 2021; Harrison and Bailey-Hytholt, 2025). These approaches should be complemented by suitable pregnancy models *in vivo*. Hamsters are well-established models of severe leptospirosis, and experimental infection of pregnant hamsters has previously demonstrated maternal and fetal injury (Sapp et al., 1980; Gomes-Solecki et al., 2017). Guinea pigs may also represent a valuable model because their placentation shares important features with humans, especially in terms of trophoblast invasion (Mess et al., 2007). Further development of pregnancy models will be important to distinguish mechanisms responsible for maternal disease and placental injury from those that promote fetal transmission. Longitudinal clinical studies are also needed to identify and validate biomarkers associated with adverse pregnancy outcomes. Integrated assessment of leptospiral burden together with markers of endothelial dysfunction, platelet and coagulation abnormalities, inflammation, and placental dysfunction may help identify pregnancies at increased risk of fetal loss, preterm birth, fetal growth restriction, or congenital infection.

From a clinical perspective, leptospirosis remains treatable with commonly used antibiotics, including ampicillin and penicillin, with no adverse effects on pregnant individuals (Selvarajah et al., 2021). However, early treatment is critical to prevent systemic dissemination and severe complications. This highlights the need to strengthen clinical and epidemiological surveillance and control systems, particularly in endemic regions. Characterization of circulating *Leptospira* still relies heavily on traditional serotyping methods, which are labor-intensive, costly, and often restricted to reference laboratories. Although efforts to implement molecular typing approaches have advanced (Cosate et al., 2017; Bilung et al., 2018; Varni et al., 2018), progress has been hindered by the complex genetic basis of *Leptospira* serovar diversity. Emerging strategies based on the presence or absence of defined gene sets offer promising alternatives for identifying circulating subtypes and improving disease control (Nieves et al., 2023).

The limited cross-protection provided by current vaccines highlights the need for strategies capable of inducing broader immunity (Grassmann et al., 2017). Interestingly, recent evidence suggests that exposure to saprophytic *Leptospira* may provide heterologous protection against subsequent pathogenic infection. Previous studies had already shown that *L. biflexa* induces a distinctive early immune response dominated by innate immune cells, with no detectable T cell activation (Shetty et al., 2021). Subsequent work in the same susceptible mouse model showed that prior exposure to live *L. biflexa* mitigated disease severity and resulted in 75% survival following challenge with *L. interrogans*, despite not preventing bacterial dissemination or renal colonization (Kundu et al., 2024). This protection was associated with increased CD4^+^ T cell and IgG2a responses, consistent with a Th1-biased adaptive response (Kundu et al., 2024). This is further supported by *in vitro* studies showing that non-pathogenic *L. biflexa* and *L. meyeri* promoted greater dendritic cell maturation and subsequent CD4^+^ T cell proliferation, including IFN-γ-producing T cells, whereas pathogenic *L. interrogans* impaired dendritic cell maturation and favored Treg induction (Krangvichian et al., 2023). Together, these findings suggest that exposure to saprophytic *Leptospira* may prime immune responses that modify subsequent responses to pathogenic infection, with potential implications for the development of broader protective vaccines. However, whether this protection extends across different pathogenic serovars remains to be established.

Beyond vaccination, effective leptospirosis control requires coordinated strategies that address human, animal, and environmental sources of transmission. These broader priorities align with the framework outlined by the Leptospirosis Burden Epidemiology Reference Group (LERG), convened by the World Health Organization (WHO) (World Health Organization, 2011). In its 2011 report, LERG identified major gaps in scientific knowledge and issued recommendations based on a multidisciplinary human-animal-ecosystem perspective. Key priorities included knowledge translation, the generation of large-scale epidemiological studies, the establishment of biobanks, and the development of expert medical centers to strengthen the prevention and control of leptospirosis.

Finally, clinical protocols should be standardized and regularly updated to facilitate timely diagnosis and intervention. These should include clinical guidelines, validated laboratory tests, well-defined differential diagnostic criteria, and structured epidemiological questionnaires to improve risk stratification and enable earlier recognition of leptospirosis in pregnant patients, ultimately improving maternal and fetal outcomes.

## 7 Conclusion

Epidemiological, clinical, and pathological evidence underscores leptospirosis as a multifaceted maternal health threat whose impact is amplified by environmental vulnerability, socioeconomic inequity, and gaps in diagnostic capacity. In pregnancy, leptospiral infection may promote systemic endothelial dysfunction, inflammation-driven vasculopathy, and placental malperfusion, pathogenic mechanisms that overlap with those implicated in human PE. This mechanistic convergence may contribute to the PE-like phenotype documented in severe cases and further highlights the clinical importance of timely recognition and treatment. Because effective antibiotic therapy is available and safe in pregnancy, improving early diagnosis, surveillance, and prevention could substantially reduce maternal and fetal morbidity. Ultimately, addressing the intertwined human, animal, and ecological dimensions of leptospirosis through a coordinated One Health framework is essential to mitigate its burden and to protect vulnerable populations, including pregnant individuals at risk of severe pregnancy complications.
